# Optical Coherence Tomography Findings in the Optic Disc Before and After Removal of Brain Tumor in a Case of Foster Kennedy Syndrome

**DOI:** 10.1155/crop/7654342

**Published:** 2026-05-07

**Authors:** Yosuke Ueno, Shigeru Honda

**Affiliations:** ^1^ Department of Ophthalmology and Visual Sciences, Osaka Metropolitan University Graduate School of Medicine, Asahimachi, Abeno-ku, Osaka, Japan, osaka-cu.ac.jp

## Abstract

We report a case of Foster Kennedy Syndrome (FKS) caused by a frontal lobe tumor in which the optic disc (OD) shape was first monitored by optical coherence tomography (OCT) before and after tumor removal. A 56‐year‐old male complained of decreased vision in the left eye and an abnormal visual field in both eyes. The OD showed swelling and redness in the right eye and paleness in the left eye. OCT was used to measure the thickness of the retinal nerve fiber layer (RNFL) and the optic nerve head (ONH). The average OD thickness in the right and left eyes was 647 and 335 *μ*m, respectively. Goldmann perimetry revealed an enlarged Mariotte blind spot with paracentral scotoma in the right eye and central absolute scotoma with horizontal hemianopsia in the left eye. FKS was suspected, and MRI revealed a lobulated mass outside the brain parenchyma of the anterior base of the skull, severely compressing both the frontal lobes and the corpus callosum. The patient underwent surgery for tumor removal. Six months after surgery, the visual field improved, but a central scotoma remained in the left eye. The OD thickness in the right and left eyes was 360 and 279 *μ*m, respectively. RNFL thickness decreased preoperatively in both eyes, reaching the atrophic range in the left eye. In conclusion, OCT analysis revealed edema in the swollen OD and in the atrophic one in FKS. Notably, the ONH may be within the normal range in the early stages of the disease.

## 1. Introduction

Foster Kennedy Syndrome (FKS) is a relatively rare disease caused by a brain tumor that presents with optic disc (OD) atrophy in one eye and OD swelling in the other, resulting in asymmetric visual acuity and visual field impairment [1]. In general, in FKS, the atrophied OD is no longer able to produce OD edema; therefore, only contralateral OD edema is observed [2]. Here, we report a case of FKS caused by a frontal lobe tumor, which was discovered with the main complaint of decreased visual acuity. We first monitored morphological changes of the OD using optical coherence tomography (OCT) before and after tumor removal.

## 2. Case Presentation

**Figure 1:**
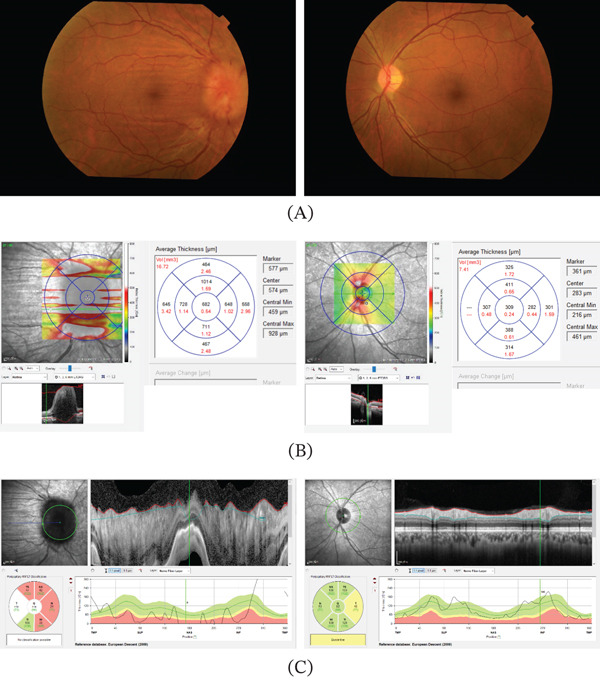
Clinical findings at the initial presentation. (A) Fundus photographs show optic disc edema in the right eye and atrophy of the left eye. (B) OCT of the right eye shows severe edema, while that of the left eye shows slight edema in the upper and lower sections. (C) In the ONH map, swelling of the right eye precluded accurate measurement, but the map of the left eye shows an almost normal range. Abbreviations: OCT, optical coherence tomography; ONH, optical nerve head.

A 56‐year‐old male presented with the chief complaint of decreased vision in the left eye and an abnormal visual field in both eyes. He had a medical history of prostatic hyperplasia but no history of allergies. The patient had no history of alcohol consumption or smoking. He gradually noticed a decrease in vision in his left eye for 10 months before the first presentation to the doctor, which had worsened, and he visited a local ophthalmology clinic. He had severe visual disturbance in the left eye (0.1 or less with Landolt C chart), visual field impairment in both eyes, and OD swelling in the right eye. Four days later, he was referred to our department to undergo further examination and treatment of suspected optic neuritis. At the time of the initial visit to our department, the findings were RV = 0.1 (1.0 × S + 2.75D = C − 0.75D A165); LV = 0.01 (n.c); RT = 11 mmHg; LT = 13 mmHg; critical flicker fusion frequency: R = 24 Hz, L = unmeasurable. Relative afferent papillary defect was positive in the left eye. No notable abnormalities were observed in the anterior segment of the intermediate translucent zone. Fundus examination revealed OD swelling and redness in the right eye and a pale OD in the left eye (Figure 1A).

**Figure 2:**
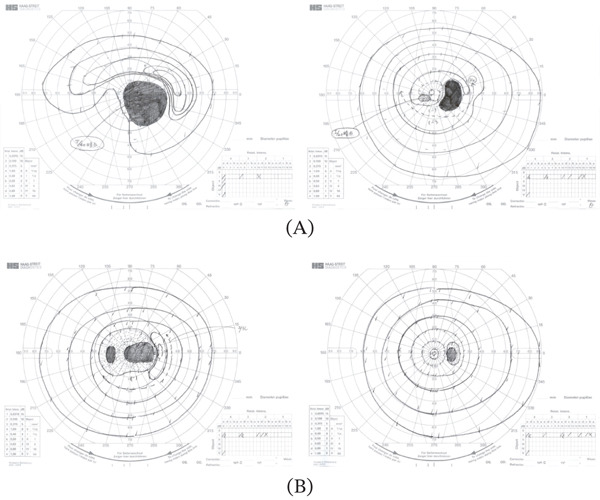
Results of Goldmann perimetry. (A) At the initial presentation, the left eye had a large central visual field loss and hemianopsia in the lower left quarter, while the right eye had an enlarged Mariotte blind spot and a paracentral scotoma in the lower left quarter. (B) At 6 months post‐operation, the central scotoma in the left eye has improved but remains, while the visual field in the right eye is almost normal.

OCT was used to evaluate the fundus status. Images were taken from the macula to the OD using an OCT‐S1 (Canon Inc., Tokyo, Japan). No notable abnormalities were found in the macula, and the OD was larger in the right eye than in the left eye; however, OD edema was observed in both eyes. The Spectralis (Heidelberg Engineering Inc., Heidelberg, Germany) was used to analyze the retinal nerve fiber layer (RNFL) thickness and measure the actual thickness of the OD swelling using the optic nerve head (ONH) program [3]. Massive OD edema was found in the right eye (Figure 1B), and RNFL thickening owing to edematous changes was also observed in the ONH (Figure 1C). The RNFL thickness in the left eye was relatively within the normal range. The mean thickness of papilledema was measured at five points: central, superior, temporal, inferior, and nasal; the right eye had a mean thickness of 682, 1014, 728, 711, and 648 *μ*m, and the left eye had a mean thickness of 309, 411, 282, 388, and 307 *μ*m, respectively. Goldmann perimetry revealed an enlarged Mariotte blind spot and paracentral scotoma in the right eye and central absolute scotoma and horizontal hemianopsia in the left eye (Figure 2A).

**Figure 3:**
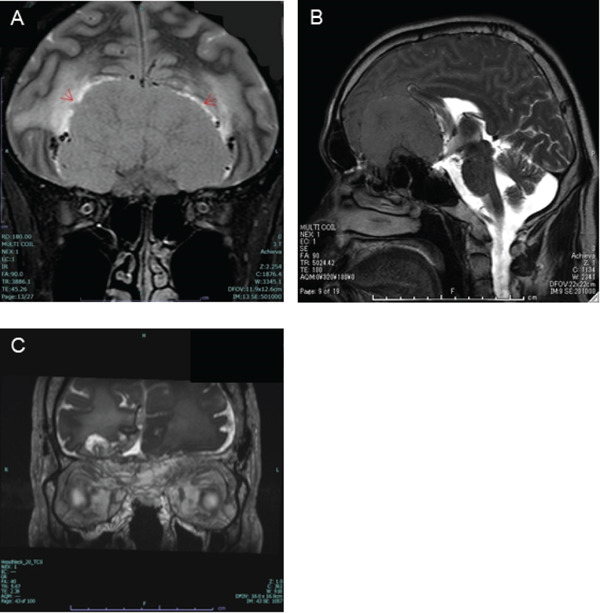
Findings on brain MRI. (A, B) At the initial presentation, a lobulated tumor measuring 7 × 6 × 4.5 cm (arrow) was found outside the brain parenchyma of the anterior base of the skull, severely compressing both frontal lobes and the corpus callosum. The tumor is isointense with the cerebral cortex on T1/T2WI and has no diffusion restriction. There are numerous flow voids at the edge and inside of the tumor. There is no noticeable compression of the optic chiasm. (C) At 3 months post‐operation, most tumor was removed, with no signs of increased intracranial pressure. A small amount of tumor remains. Abbreviations: MRI, magnetic resonance imaging; WI, weighted image.

The examination also revealed olfactory disturbances and bladder–rectal disorders. Based on the typical difference between the left and right ODs and the shape of the visual field disorder, FKS was suspected, and brain contrast MRI was performed. The results showed a lobulated mass measuring 7 × 6 × 4.5 cm outside the brain parenchyma at the anterior base of the skull, severely compressing both the frontal lobes and corpus callosum (Figure 3A, B).

**Figure 4:**
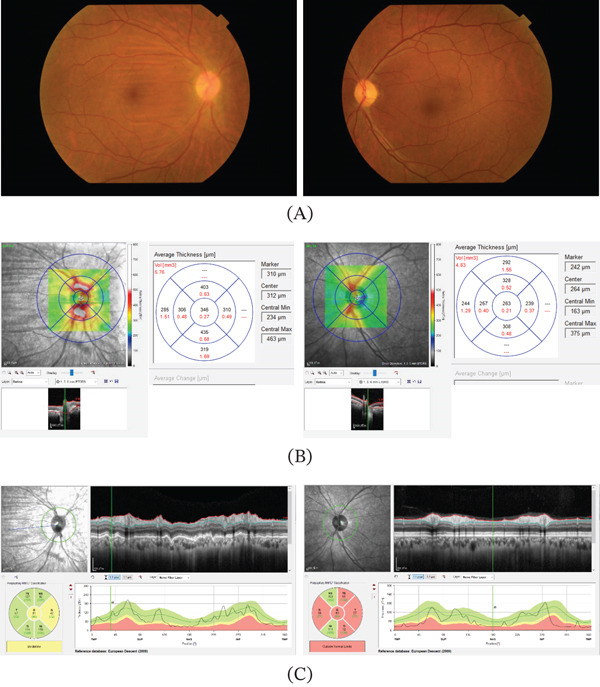
Clinical findings at 6 months post‐operation. (A) Improvement in optic disc edema and some retinal walls are found in the right eye, while there is a progressive optic disc atrophy in the left eye. (B) OCT shows improvement in optic disc edema. (C) In the ONH map, the right eye shows a nearly normal RNFL thickness, while the left eye has thinning of the RNFL thickness all around. Abbreviations: OCT, optical coherence tomography; ONH, optical nerve head; RNFL, retinal nerve fiber layer.

The tumor was isodense in the brain cortex on both T1‐ and T2‐weighted images (WI), and no diffusion restriction was observed. Numerous flow voids were observed at the edge and inside the tumor, and no significant compression was observed at the optic chiasm. Based on these findings, the patient was diagnosed with visual disturbances and FKS caused by an intracranial tumor, and the neurosurgery department was consulted. The patient was immediately admitted to the hospital and underwent surgery to remove the tumor. Surgery was preceded by intratumoral vascular embolization, and a transnasal endoscopy and craniotomy were performed for tumor removal. The pathological and clinical diagnoses were meningothelial meningioma (MGM) and planum sphenoidale MGM, respectively. Three months after the surgery, the OD edema in the right eye subsided, and the OD atrophy in the left eye progressed. The visual field showed improvement in the scotoma in both eyes, and visual acuity improved to (1.2) in the right eye; however, vision in the left eye (0.01) remained poor. MRI showed that the tumor had been almost completely resected, and signs of increased intracranial pressure had improved (Figure 3C). Six months after the surgery, ophthalmoscopy revealed improvement in the OD of the right eye, whereas atrophic changes in the OD of the left eye progressed (Figure 4A).

The visual field showed further improvement; however, the central scotoma in the left eye persisted (Figure 2B), and the visual acuity was 1.2 in the right eye and 0.02 in the left eye. OCT topography analysis of the OD using OCTS1 showed a reduction in OD edema in both eyes with no retinal circulatory disorders. Using Heidelberg Spectralis, the mean peripapillary thickness was 346, 403, 306, 435, and 310 *μ*m in the right eye, and 263, 328, 239, 308, and 257 *μ*m in the left eye (Figure 4B). In the ONH analysis, the RNFL thickness also decreased preoperatively in both eyes. In the center, above, below, on the temporal side, below and on the nasal side, the changes were −336, −611, −422, −276, and −338 *μ*m (average −396 *μ*m) in the right eye and −46, −83, −43, −80, and −50 *μ*m (average −60 *μ*m) in the left eye, respectively. In the left eye, the lesion descended to an atrophic zone (Figure 4C). Although bladder and rectal dysfunction improved, olfactory impairment persisted.

## 3. Discussion

We report a case of FKS caused by a frontal lobe tumor. Qualitative and quantitative evaluations of the OD and peripapillary retinal thickness using OCT revealed that edema reactions were also observed in atrophic eyes in FKS. Notably, the ONH was within the normal range at the first consultation.

FKS is characterized by OD atrophy on the affected side and OD swelling on the contralateral side caused by an intracranial mass. OD atrophy on the affected side is due to compression, whereas OD edema on the contralateral side is due to secondary intracranial hypertension. Each presents with a different visual field disorder. Its etiology involves intracranial tumors, particularly those on the underside of the frontal lobe. Olfactory sulcus and sphenoid wing meningiomas are common, and olfactory abnormalities can occur because of olfactory nerve compression [4]. As shown in the present case, planum sphenoidale meningiomas are slow‐growing extracerebral tumors that arise from the area between the roof of the sphenoid sinus, optic nerve, and anterior clinoid process. Swelling of the meningioma usually compresses the optic nerve dorsally and caudally, causing vision loss as the main symptom [5]. Sphenoid meningiomas account for 5%–10% of intracranial meningiomas and are more prevalent in women [6]. Most cases have been reported in adults, with a predominance in patients aged > 65 years [7]. Because the disease progresses slowly and symptoms appear subtly over a long period, there is a high tendency for delayed diagnosis [7]. In this case, the meningioma was quite large, but the patient only experienced visual symptoms in the left eye, whereas the right eye was normal; therefore, it was left untreated. However, visual field testing revealed abnormalities in the right eye. This was due to the large lesion compressing the optic nerve of the left eye, which caused increased intracranial pressure and OD edema in both eyes. The optic nerve is typically located near the anterior olfactory sulcus. Olfactory groove meningiomas and sphenoid sinus squamous meningiomas often show symptoms such as dementia, personality changes, and olfactory disorders; however, these symptoms improve after tumor removal [8]. In the present case, no cognitive or personality changes were observed; however, the patient presented with an olfactory disorder. OCT confirmed swelling of the OD in both eyes and reduction in edema before and after excision surgery. In contrast, OCTA showed no abnormalities or changes in retinal circulation before and after surgery. It has been reported that the ONH thickens in cases of OD edema caused by increased intracranial pressure [9]. The exact cause is unknown; however, increased intracranial pressure has been shown to cause increased pressure in the optic nerve sheath, which stagnates axonal blood flow and reduces blood flow to axons in the nerve canal. Papilledema occurs as a result of the axonal expansion of the prelaminar and peripapillary nerve fibers. After acute axonal blood flow stagnation, vascular congestion, leakage, and ischemia occur, accompanied by interstitial edema, which is thought to lead to RNFL thickening [10]. After tumor removal, a reduction in OD edema was observed. The OD of the left eye showed signs of atrophy; however, papilledema was observed. Edema can occur even if the disc is atrophied. The change in the right eye was papilledema caused by increased intracranial pressure, while the change in the left eye was due to both direct compression of the tumor and increased intracranial pressure. The former causes atrophy of the optic nerve fibers, and the latter causes hypertension, resulting in different degrees of edema between the left and right eyes [11]. In this case, the right eye had severe OD swelling and a thick RNFL, whereas the left eye had normal thickness at the time of the initial examination. After tumor removal, the RNFL thickness of the right eye returned to near normal, whereas that of the left eye narrowed and thinned. At the initial consultation, the OD of the left eye was originally prone to atrophy. However, owing to the effects of OD edema caused by increased intracranial pressure, it became near normal in thickness. After surgery, the increased intracranial pressure was relieved, and the edema improved, resulting in a normal thickness range in the right eye and thinning in the left eye because of atrophy. The thinning was particularly noticeable on the temporal side. This is presumed to be a result of a central visual field disorder in the left eye and progressive atrophy, especially on the temporal side. In this case, 11 months had passed from the awareness of decreased vision in the left eye to tumor removal, and atrophy of the OD had progressed. However, visual function improved only to a limited extent after tumor removal. Paradoxically, this suggests that a state in which edema can occur may still be one in which improvement of nerve fibers can be expected.

As a limitation of this study, it is the first report of FKS in which ODs were monitored by OCT. Hence, our findings should be replicated in other FKS cases to be warranted.

In conclusion, OCT may reveal edema in the swollen OD and in the atrophic one in FKS. The ONH may be within the normal range in the early stages of the disease.

## Author Contributions

Yosuke Ueno collected the data and drafted the manuscript. Shigeru Honda interpreted the data and critically reviewed the manuscript.

## Funding

No funding was received for this manuscript.

## Disclosure

Both authors read and approved the final manuscript.

## Ethics Statement

This study was conducted according to the principles of the Declaration of Helsinki. Written informed consent was obtained from all participants for the publication of case reports and accompanying images. Ethical approval was not required for this study in accordance with local or national guidelines.

## Conflicts of Interest

The authors declare no conflicts of interest.

## Data Availability

All data generated or analyzed during this study are included in this article. Further inquiries can be directed to the corresponding author.
